# Impact of faculty and resident gender on milestone evaluations in anesthesiology residency: a retrospective analysis

**DOI:** 10.1080/10872981.2026.2688660

**Published:** 2026-07-10

**Authors:** Yvette Martin McGrew, Darrell R. Schroeder, Steven H. Rose, Bhargavi Gali

**Affiliations:** a Department of Anesthesiology and Perioperative Medicine, Mayo Clinic, Rochester, MN, USA; b Department of Health Sciences Research, Mayo Clinic, Rochester, MN, USA

**Keywords:** Gender bias, anesthesiology, residency, qualitative, evaluation

## Abstract

**Background:**

Anesthesia-specific milestone evaluations are intended to objectively assess performance in ACGME core competencies for anesthesiology residents. Recent reports in several medical specialties have documented differences in resident evaluations based on gender.

**Objective:**

We studied the impact of faculty and resident gender on milestone evaluations during Anesthesiology residency.

**Design:**

This was a retrospective analysis of 20,570 anesthesiology resident milestone evaluations from a single institution over a 7-year period during the ACGME milestones 1.0 time frame. Standardized scores were analyzed using a linear mixed model.

**Results:**

170 anesthesiology residents were evaluated by 194 faculty. For CA1 evaluations, mean standardized milestone scores for the core competencies of professionalism (*p* = 0.002) and interpersonal/communication (*p* < 0.001) and for overall assessment (*p* = 0.009) were dependent on faculty gender. For CA2 evaluations, regardless of the evaluator gender, the mean standardized milestone scores for many individual core competencies were significantly higher for female residents compared to male residents. For CA3 evaluations, mean standardized milestone scores did not differ significantly between male residents and female residents for any core competency, however overall assessment scores were dependent on faculty gender with male faculty scoring male residents more highly and female faculty scoring female residents more highly (*p* < 0.001).

**Conclusions:**

We observed an association between faculty gender and milestone evaluation scores of residents that was most evident for CA1 residents. We also observed the overall assessment scores for male and female residents were significantly influenced by the gender of the faculty evaluator. Further work is required to better understand and mitigate gender bias to promote equity in resident assessment.

## Introduction

Valid and meaningful assessment of residents and fellows provides a foundation that impacts Graduate Medical Education (GME) training programs, resident and fellow physician advancement, career opportunities, and patient care. GME training programs use a variety of tools to assess resident performance. These assessments determine resident progress toward independent practice, advancement in training level, and document competence. Therefore, it is important to ensure these high-stakes assessments are fair, thorough, and free of bias.

The Accreditation Council for Graduate Medical Education (ACGME) established six core competency categories to assess residents and fellows – patient care, medical knowledge, professionalism, interpersonal and communication skills, practice-based learning and improvement, and systems-based practice [1] (Table 1). In 2014, anesthesiology educational milestones were established by the Anesthesiology Milestone Working Group to improve the assessment of anesthesiology residents in the six ACGME core competency categories [2]. Quantitative assessment of these milestones for anesthesia resident physicians is an essential component of anesthesiology medical education [3,4]. Applying numeric values to milestone assessment provides a measure to track progress within the ACGME core competency categories throughout training. This approach not only provides a clear and objective measure of their development but also ensures continual monitoring and support of their educational growth throughout training [5].

**Table 1.** ACGME anesthesiology core competencies [1].

Core competencies
Patient care
Medical knowledge
Practice based learning
Professionalism
Interpersonal
Systems based practice

Gender-based differences in milestone evaluations of residents have recently been documented in other medical specialties such as emergency medicine and internal medicine [6–8]. A literature review of residency evaluations found both significant differences in narrative assessments and performance metrics. This included a gender associated lack of consistency of feedback [9]. Additionally, the review suggested that there is a potential role of gender of faculty in resident assessments. They noted a trend towards male faculty rating male residents higher. Another study looking specifically at internal medicine residents found comments for female residents tended to be less specific for areas of growth, or there was more likely to be no comments [10].

Gender differences in resident evaluations are driven by subjective and structural differences in feedback leading to consistently lower ratings for females despite similar performance [11,12]. Although the number of studies addressing gender-based differences in evaluations in most specialties is increasing [13–15], limited data exist that address the effect of gender on milestone evaluations in anesthesiology [16].

We studied differences in milestone evaluation scores within each ACGME core competency category between male and female anesthesiology residents in a single program to determine the impact of faculty and resident gender on anesthesiology resident milestone evaluations completed by faculty. Identifying the influence of gender on resident evaluations in anesthesiology is important to ensure residents are evaluated fairly and to ensure advancement and career opportunities are merit-based [17,18].

## Materials and methods

### Study design

We conducted a retrospective analysis of resident milestone evaluations from a single United States Anesthesiology residency program. Our study was reviewed and approved by the Mayo Clinic Institutional Review Board (IRBe) ID: 20-003747. The Mayo Clinic Institutional Review Board (IRB) waived the requirement for written informed consent for this study. The research design strictly adheres to the ethical principles of the ‘Helsinki Declaration’. Research data were collected from the GME management system (MedHub LLC, Michigan) from July 2014 to August 2020 during the ACGME milestones 1.0 timeframe. Faculty and Resident gender were self-reported at the time of appointment. At the time of matriculation, the gender question only allowed respondents to select ‘male’ or ‘female’ with no other options for gender identity. Individual faculty assessment from each faculty member who had worked with each resident was obtained after each clinical rotation. Evaluations during away rotations were not included, as faculty on the away rotations were not members of our department and their practice was dissimilar to internal faculty practice. On call experiences were not included, as the evaluation form for on call evaluations was created specifically for on call responsibilities. All evaluations were recorded using a quantitative scoring system. Each numeric assessment was on a scale of 1 to 9. An example evaluation has been included in the supplemental material. Data were deidentified and stratified by a neutral analyst according to resident and faculty gender to create a complete data set. Anesthesia-specific milestone assessment data included all core competency categories (patient care, medical knowledge, systems-based practice [SBP], practice-based learning and improvement [PBLI], professionalism, and interpersonal and communication skills [ICS]). Evaluation data for the patient care subcategory, technical skills, was also included. In addition to milestone scores within each of the core competencies, each evaluation contained an overall score that was completed by the faculty. This overall assessment score was also included.

### Statistical analysis

Standardized milestone scores were analyzed using linear mixed-effects models, with crossed resident-specific and evaluator-specific random intercepts included to account for residents being assessed by multiple evaluators and evaluators assessing multiple residents. The fixed effects of interest were resident gender, evaluator gender, and clinical anesthesia (CA) year. Including resident gender and evaluator gender as fixed effects explicitly models the effects of gender, while the random intercepts account for individual-level variation that is independent of the group-level gender effects. Initial analyses were performed to assess CA year-specific differences between male and female residents, and CA year-specific differences between male and female evaluators. A final comprehensive model was fit which included resident gender, evaluator gender, CA year, and all two-way and three-way interaction effects. Linear contrasts were used to estimate mean (SE) milestone scores for male and female residents according to CA year and evaluator gender, along with *p*-values for the resident gender by evaluator gender interaction effect. Separate analyses were performed for each of the 6 ACGME core competency categories, the patient care subcategory technical skills and for the overall assessment. In all cases, model convergence was attained using the default relative Hessian convergence criterion (1 × 10^−8^) and variance components were estimated for the resident and evaluator random intercepts (Supplemental Table). Recognizing that multiple correlated outcomes were assessed, two-tailed *p* < 0.01 was used to denote statistical significance, and results were interpreted based on the overall pattern and consistency of findings. All analyses were performed using SAS, version 9.4 (SAS Institute, Inc; Cary, NC).

## Results

This investigation includes data from anesthesia resident evaluations completed between July 2014 and August 2020. Our study included 170 anesthesiology residents (85 males and 85 females) who were evaluated by 194 faculty evaluators (144 male, 50 female). The median (25th, 75th) number of faculty evaluating each resident was 62 (49, 73) during CA1, 55 (39, 69) during CA2 and 57 (23, 75) during CA3. Additional details regarding the gender breakdown of evaluations are presented in Table 2.

**Table 2.** Faculty and resident characteristics^a^.

Characteristic	Overall	Clinical anesthesia year		
		CA1	CA2	CA3
Faculty evaluators				
* N*	194	182	183	186
Male, *n* (%)	144 (74)	136 (75)	135 (74)	139 (75)
Female, *n* (%)	50 (26)	46 (25)	48 (26)	47 (25)
Residents				
* N*	170	131	126	120
Male, *n* (%)	85 (50)	66 (50)	63 (50)	58 (48)
Female, *n* (%)	85 (50)	65 (50)	63 (50)	62 (52)
Evaluations per resident				
Overall				
All evaluators	121 (64, 178)	62 (49, 73)	55 (39, 69)	57 (23, 75)
Male evaluators	96 (48, 139)	49 (39, 61)	42 (31, 54)	44 (20, 60)
Female evaluators	29 (18, 41)	13 (10, 16)	14 (10, 17)	13 (10, 17)
Male residents				
All evaluators	121 (67, 176)	61 (47, 71)	52 (37, 65)	65 (49, 78)
Male evaluators	97 (53, 134)	49 (36, 59)	39 (28, 49)	52 (37, 63)
Female evaluators	27 (19, 41)	13 (10, 15)	14 (10, 16)	14 (12, 18)
Female residents				
All evaluators	120 (58, 181)	62 (51, 75)	58 (39, 80)	48 (9, 66)
Male evaluators	94 (44, 144)	50 (39, 61)	46 (31, 61)	34 (8, 49)
Female evaluators	31 (18, 41)	12 (10, 17)	14 (10, 21)	12 (4, 15)

^a^ Data are summarized using *n* (%) or median (25th, 75th).

### Influence of resident gender on scores

We first compared the milestone scores of male and female residents independent of evaluator gender. Milestone scores for the six core competency categories, as well as the Patient Care subcategory technical skills and an overall assessment score are presented in Table 3 according to resident gender and CA year. Both genders show progression in milestone scores through CA1 to CA3 for each competency category. There is no difference in scores between genders for the CA1 year. However, there is a difference in scores for four out of six competency categories in the CA2 year with female residents scoring higher than male residents in Patient Care (6.76 vs 6.4 *p* < 0.001), Practice Based Learning (5.89 vs 5.55 *p* = 0.001), Professionalism (7.08 vs 6.78 *p* = 0.001), and Interpersonal/Communication (6.99 vs 6.70 *p* = 0.002). CA2 female residents have a higher milestone score than male residents in the Patient Care subcategory, technical skills (6.70 vs 6.3 *p* < 0.001). No differences are noted during the CA3 year.

**Table 3.** Mean milestone scores according to resident gender and clinical anesthesia year^a^.

	CA1	CA2	CA3
Patient care			
Male resident	4.64 (0.10)	6.40 (0.10)	7.97 (0.10)
Female resident	4.70 (0.10)	6.76 (0.10)	8.03 (0.10)
* p*-value^b^	0.524	<0.001	0.513
Patient care: technical skills			
Male resident	4.42 (0.10)	6.34 (0.10)	7.87 (0.10)
Female resident	4.45 (0.10)	6.70 (0.10)	7.96 (0.10)
* p*-value^b^	0.702	<0.001	0.280
Medical knowledge			
Male resident	4.98 (0.10)	6.45 (0.10)	7.87 (0.11)
Female resident	5.01 (0.10)	6.61 (0.10)	7.9 (0.11)
* p*-value^b^	0.705	0.073	0.787
Practice based learning			
Male resident	4.03 (0.18)	5.55 (0.18)	6.72 (0.18)
Female resident	4.10 (0.18)	5.89 (0.18)	6.61 (0.18)
* p*-value^b^	0.513	0.001	0.321
Professionalism			
Male resident	5.22 (0.12)	6.78 (0.12)	8.19 (0.12)
Female resident	5.32 (0.12)	7.08 (0.12)	8.13 (0.12)
* p*-value^b^	0.283	0.001	0.555
Interpersonal/communication			
Male resident	5.03 (0.10)	6.70 (0.10)	8.18 (0.10)
Female resident	5.13 (0.10)	6.99 (0.10)	8.27 (0.11)
* p*-value^b^	0.313	0.002	0.343
Systems based practice			
Male resident	5.02 (0.17)	6.41 (0.16)	7.69 (0.17)
Female resident	5.16 (0.17)	6.61 (0.16)	7.64 (0.18)
* p*-value^b^	0.325	0.077	0.760
Overall assessment			
Male resident	6.96 (0.10)	7.58 (0.10)	8.27 (0.10)
Female resident	6.83 (0.10)	7.67 (0.10)	8.15 (0.10)
* p*-value^b^	0.184	0.341	0.249

^a^ Data presented correspond to the estimated mean (SE) milestone score obtained from a linear mixed model which included resident gender and clinical anesthesia year (CA1, CA2, CA3) as fixed effects along with resident-specific and evaluator-specific random intercepts.

^b^ *p*-value for the comparison of the estimated milestone score between male and female residents.

Overall assessment scores are also presented in Table 3. Both genders show progression in the overall assessment score through CA1 to CA3. There is no statistical difference between genders in overall assessment score for any CA group.

### Influence of faculty evaluator gender on scores

To further investigate gender influence on scores, we then compared milestone scores from male and female faculty evaluators independent of resident gender as presented in Table 4. Male faculty evaluators assigned higher milestone scores compared to female faculty evaluators for the competency Systems Based Practice in the CA1 year (5.30 vs 4.32, *p* = 0.006) and for Medical Knowledge in the CA2 year (6.67 vs 6.16, *p* = 0.008). There were no statistically significant differences in milestone scoring between male and female faculty evaluators in CA3 year for any competency.

**Table 4.** Mean milestone scores according to faculty evaluator gender and clinical anesthesia year^a^.

	CA1		CA2		CA3	
	Male evaluator	Female evaluator	Male evaluator	Female evaluator	Male evaluator	Female evaluator
Patient care	4.76 (0.10)	4.36 (0.16)	6.69 (0.10)	6.26 (0.16)	8.05 (0.10)	7.88 (0.17)
* p*-value^b^	0.032		0.018		0.364	
Patient care: technical skills	4.47 (0.10)	4.32 (0.17)	6.63 (0.10)	6.25 (0.17)	7.95 (0.10)	7.83 (0.17)
* p*-value^b^	0.447		0.046		0.541	
Medical knowledge	5.10 (0.11)	4.67 (0.17)	6.67 (0.11)	6.16 (0.17)	7.93 (0.11)	7.79 (0.17)
* p*-value^b^	0.026		0.008		0.482	
Practice based learning	4.14 (0.20)	3.78 (0.34)	5.77 (0.20)	5.59 (0.34)	6.76 (0.20)	6.39 (0.34)
* p*-value^b^	0.354		0.637		0.340	
Professionalism	5.30 (0.12)	5.12 (0.21)	6.95 (0.12)	6.87 (0.20)	8.19 (0.13)	8.14 (0.21)
* p*-value^b^	0.423		0.758		0.842	
Interpersonal/communication	5.17 (0.10)	4.80 (0.17)	6.95 (0.10)	6.54 (0.17)	8.26 (0.10)	8.15 (0.17)
* p*-value^b^	0.044		0.025		0.525	
Systems based practice	5.30 (0.17)	4.32 (0.31)	6.65 (0.17)	6.05 (0.30)	7.72 (0.17)	7.63 (0.32)
* p*-value^b^	0.006		0.075		0.787	
Overall assessment	6.90 (0.10)	6.86 (0.15)	7.66 (0.10)	7.54 (0.15)	8.24 (0.10)	8.12 (0.15)
* p*-value^b^	0.819		0.454		0.461	

^a^ Data presented correspond to the estimated mean (SE) milestone score obtained from a linear mixed model which included evaluator gender and clinical anesthesia year as fixed effects along with resident-specific and evaluator-specific random intercepts.

^b^ *p*-value for the comparison of the estimated milestone score between male and female evaluators.

### Interaction of faculty evaluator gender and resident gender on scores

After observing the influence of resident gender or faculty evaluator gender on milestone scores independently, we tested the potential for any interaction of faculty gender and resident gender on milestone scores for each competency category and the overall score. Results are presented in Table 5 according to resident gender, faculty gender, and CA year. Evaluation of CA1 residents by male faculty revealed no significant differences in mean standardized milestone scores between male and female residents. However, for CA1 residents mean standardized milestone scores by female faculty were significantly higher for female residents in the competency categories professionalism (4.89 vs 5.31, *p* = 0.002) and interpersonal/communication skills (4.61 vs 4.97, *p* < 0.001). For CA2 evaluations, both male and female faculty assigned higher mean standardized milestone scores to female residents compared to male residents across all core competencies. However, the difference in milestone scores assigned by male faculty to male and female residents compared to the difference in scores assigned by female faculty to male and female residents was not statistically significant for any of the core competencies, indicating a similar scoring pattern for residents regardless of the faculty's gender. For CA3 evaluations, the mean standardized milestone scores from male or female faculty evaluators did not differ significantly between male residents and female residents for any core competency.

**Table 5.** Mean milestone scores according to resident gender, faculty evaluator gender, and clinical anesthesia year^a^.

	CA1		CA2		CA3	
	Male evaluator	Female evaluator	Male evaluator	Female evaluator	Male evaluator	Female evaluator
Patient care						
Male resident	4.76 (0.11)	4.28 (0.17)	6.52 (0.11)	6.05 (0.17)	8.04 (0.11)	7.79 (0.17)
Female resident	4.78 (0.11)	4.46 (0.17)	6.86 (0.11)	6.45 (0.17)	8.05 (0.11)	7.98 (0.17)
Interaction *p*-value^b^	0.043		0.452		0.054	
Patient care: technical skills						
Male resident	4.47 (0.11)	4.27 (0.18)	6.46 (0.11)	6.02 (0.18)	7.91 (0.11)	7.75 (0.18)
Female resident	4.48 (0.11)	4.39 (0.18)	6.78 (0.11)	6.45 (0.18)	7.98 (0.11)	7.92 (0.18)
Interaction *p*-value^b^	0.205		0.209		0.311	
Medical knowledge						
Male resident	5.11 (0.12)	4.57 (0.18)	6.60 (0.11)	6.05 (0.18)	7.95 (0.12)	7.68 (0.18)
Female resident	5.11 (0.12)	4.76 (0.18)	6.74 (0.12)	6.27 (0.18)	7.89 (0.12)	7.92 (0.19)
Interaction *p*-value^b^	0.045		0.328		0.015	
Practice based learning						
Male resident	4.16 (0.21)	3.61 (0.35)	5.60 (0.21)	5.42 (0.35)	6.82 (0.21)	6.35 (0.35)
Female resident	4.15 (0.21)	3.95 (0.35)	5.94 (0.21)	5.77 (0.35)	6.67 (0.21)	6.42 (0.35)
Interaction *p*-value^b^	0.040		0.912		0.227	
Professionalism						
Male resident	5.30 (0.13)	4.89 (0.22)	6.81 (0.13)	6.69 (0.21)	8.22 (0.13)	8.10 (0.22)
Female resident	5.33 (0.13)	5.31 (0.22)	7.09 (0.13)	7.05 (0.21)	8.12 (0.14)	8.16 (0.22)
Interaction *p*-value^b^	0.002		0.449		0.260	
Interpersonal/communication						
Male resident	5.17 (0.11)	4.61 (0.18)	6.80 (0.11)	6.39 (0.17)	8.23 (0.12)	8.03 (0.18)
Female resident	5.19 (0.11)	4.97 (0.18)	7.09 (0.11)	6.68 (0.17)	8.28 (0.12)	8.27 (0.18)
Interaction *p*-value^b^	<0.001		0.985		0.068	
Systems based practice						
Male resident	5.29 (0.19)	3.93 (0.35)	6.56 (0.18)	5.95 (0.32)	7.77 (0.19)	7.47 (0.34)
Female resident	5.32 (0.18)	4.59 (0.33)	6.76 (0.18)	6.13 (0.32)	7.64 (0.19)	7.78 (0.35)
Interaction *p*-value^b^	0.024		0.918		0.097	
Overall assessment						
Male resident	6.99 (0.11)	6.85 (0.16)	7.62 (0.11)	7.47 (0.16)	8.33 (0.11)	8.08 (0.16)
Female resident	6.82 (0.11)	6.87 (0.16)	7.70 (0.11)	7.60 (0.16)	8.14 (0.11)	8.18 (0.16)
Interaction *p*-value^b^	0.009		0.513		<0.001	

^a^ Data presented correspond to the estimated mean (SE) milestone score for each competency obtained from a linear mixed model which included resident gender, evaluator gender and clinical anesthesia as fixed effects along with resident-specific and evaluator-specific random intercepts.

^b^ *p*-value for the resident-gender by evaluator-gender interaction effect assessing whether differences in milestone scores between male and female residents are dependent on the gender of the evaluator for each competency and overall assessment.

Mean standardized milestone scores from male or female faculty evaluators did not differ significantly for the Patient Care subcategory technical skills for male or female residents at any point in training.

Standardized scores for overall assessment from male faculty were higher for male residents compared to female residents (6.99 vs 6.82 *p* = 0.009) in the CA1 year. This was observed again in the CA3 year with the mean standardized scores for overall assessment from male faculty higher for male residents compared to female residents (8.33 vs 8.14 *p* < 0.001). Mean standardized scores for overall assessment from female faculty were higher for female residents compared to male residents (8.18 vs 8.08 *p* < 0.001) during CA3. Although significant for CA1 and CA3, the mean standardized score for overall assessment from male or female faculty did not differ significantly between male and female CA2 residents.

## Discussion

Our retrospective analysis of a large U.S. anesthesiology residency program documented an influence of resident and faculty gender on anesthesiology resident milestone assessment.

The three clinical anesthesia years CA1 through CA3 include training in basic, subspecialty and advanced anesthesia. The first year consists of clinical rotations in basic anesthesiology. The second and third years consist of clinical rotations in subspecialty and advanced anesthesia with more responsibility and autonomy in preparation for independent practice.

We found differences in CA1 milestone scores for the Professionalism and Interpersonal/Communication competencies are dependent on faculty gender. Both male and female faculty assign higher milestone scores to female residents than male residents for these competencies, but female faculty rated female residents significantly more highly than male faculty did. While the milestone scores for professionalism and interpersonal/communication analyzed only by resident gender did not reveal significant differences, the inclusion of both faculty gender and resident gender uncovered meaningful associations. This result demonstrates the importance of stratifying data by faculty gender to tease out potential areas of preference or bias. Professionalism and Interpersonal/Communication entails teamwork and reliability, areas where traditionally females are categorized, which can influence subjective assessment [19,20]. The higher score from both male and female faculty could suggest bias.

We found that differences in CA2 milestone scores are independent of faculty gender. Differences in milestone scores for certain competency categories based on resident gender were only observed at the CA2 level. The finding that female residents receive higher milestone scores in almost all competencies during the CA2 year of residency regardless of faculty gender is similar to findings by Klein et al in PGY2 Internal Medicine residents [7]. In contrast to four years of postgraduate anesthesiology training, internal medicine has only 3 years of postgraduate training, Both Internal Medicine PGY2 and anesthesiology CA2 years of training are associated with increasing responsibility, managing more complex patients, and becoming the first point of contact for questions about patients, and taking on a more active role in interdisciplinary teams. Therefore the Internal Medicine PGY2 year would be equivalent to an Anesthesiology CA2 year. Klein found that during the IM-PGY2 year, female residents received consistently higher competencies scores regardless of faculty gender. They provide no definitive explanation for this, but this observation may deserve further investigation since it is also observed in another specialty. Our hypothesis is related to the evaluation differences observed in the CA1 year. The higher rating for professionalism and interpersonal/communication in the CA1 evaluations suggests a bias that female residents are recognized as demonstrating compassion, responsiveness to patient needs, and working effectively as a team member within the clinical environment. We hypothesized that by the second year, when there is a constant change of specialty rotations, residents already attaining higher professionalism and interpersonal/communication scores adapt more quickly to implicit expectations of a rotation such as evaluator preferences or rotation specific expectations rather than just clinical knowledge and thus may score higher when they are expected to function more independently. By CA3 year this is normalized as both male and female residents have achieved similar levels of clinical exposure, knowledge and independence, which is supported by both Klein and our study, where residents achieve similar progression in milestone scores by the final year of training.

We also included the overall assessment in our study as it reflects a global assessment of a resident's performance. We found differences in overall assessment between male and female residents is dependent on faculty gender at the CA3 level. Intuitively, scores for overall assessment should reflect a summary of resident performance in the individual competencies. Interestingly, while there were no differences in rankings between male and female residents for individual competency categories at the CA3 level, male faculty rated male residents more highly in overall assessment and female faculty rated female residents more highly. This raises an interesting hypothesis that discrete assessments such as milestone-based core competency evaluation may be less susceptible to gender bias than global assessments on an overall assessment score. This observation that certain types of evaluations are more susceptible to gender bias has been observed in studies evaluating surgical residents [21,22].

This is the first study to examine the impact of gender on performance evaluations in a U.S. anesthesiology residency. These findings are consistent with evidence of gender influence in U.S. medical training in other specialties [23,24]. A recent large study by Matava et al showed no difference in assessment of Canadian anesthesiology residents based on resident or faculty gender [16]. However, there are notable differences in the study design. Faculty in the study by Matava et al were provided information via Grand Rounds presentations and education via educational videos and email prior to implementation of the assessment tools. Additionally, one of the tools was a daily assessment tool completed after one interaction, as opposed to our end of rotation evaluations. In our study we assessed for gender influence on milestone assessment within individual competency categories, while the study by Matava *et.al*. assessed for gender influence on aggregate data of only those evaluations that were deemed to have high competency scores. They did not assess for gender influence on individual competencies or across the spectrum of achievement. Having only assessed evaluations that have high achievement scores may have eliminated any gender bias in their data. This difference in assessment tools likely contributes to the differences in our results.

Recent literature found gender differences in resident evaluations including narrative assessments, performance scores, and inconsistent feedback [9]. A study of internal medicine narrative evaluations found that comments were either not given, or more likely to be non-specific, resulting in lost educational opportunities for female residents [10].

Addressing these issues will be important for all specialties. Potential interventions could include implementing dedicated faculty training to understand potential biases and how to recognize them [25]. Future directions could include creating processes at each institution to ensure awareness of these issues, and education for all who supervise residents, including follow up to assess impact of interventions.

Our study is limited by its observational design. The value of these observational data is the emergence of questions that provide a foundation for further investigation. These differences may be influenced by conscious or unconscious gender bias. The mechanisms of this bias and methods to reduce the influence of gender bias deserve further exploration. Current approaches within medical education to reduce bias have included faculty training on implicit bias and multiple evaluations [26]. We currently lack the methodology to determine an absolute score for male and female residents at each level to determine if there were actual performance differences or if an element of gender bias was involved. As our data come from a single large academic institution and our findings may not be generalizable, larger multi-institutional studies would help normalize these data to further understand differences that are observed.

A second limitation of our study is the data come from a single large academic institution and our findings may not be generalizable. At the time of our study, we only had the binary gender selection available from the ACGME survey and therefore we focused on male and female gender. This is a limitation of our study as it overlooks the other types of diversity that exists among residents and faculty. Future studies should include racial, ethnic, and additional gender groups. Lastly, although there are statistically significant findings, at present it is uncertain if the differences are of practical importance when evaluating residents. This is an area for future investigation.

In conclusion, assessments that are fair and free of bias are critically important to residents, programs, and the public. These findings are important because identifying and understanding these differences could reduce or eliminate bias in assessment based on gender. Our data do not support a conclusion of major gender bias in anesthesiology resident evaluations. However, our data reveal patterns of concern that deserve further exploration to eliminating gender bias in resident evaluation.

## Supplementary Material

Supplementary material Supplemental_Table1.docx

Supplementary Material supplemental material.docx

## Data Availability

Data available on request from authors. The data that support the findings of this study are available from the corresponding author, [YMM], upon reasonable request.

## Glossary of terms

ACGME: Accreditation Council for Graduate Medical Education
PGY: post-graduate year
SBP: systems-based practice
PBLI: practice-based learning and improvement
ICS: interpersonal/communication skills
CA: Clinical Anesthesia year
