# Supplementary material for: Impact of faculty and resident gender on milestone evaluations in anesthesiology residency: a retrospective analysis
Source: Med Educ Online. 2026 Jul 10;31(1):2688660. doi: 10.1080/10872981.2026.2688660 (PMC13360505; doi:10.1080/10872981.2026.2688660)
Supplement: Supplementary material — Supplemental_Table1.docx [file ZMEO_A_2688660_SM0909.docx]

Supplemental Table 1. Variance Components*

| Score | Resident (SE) | Evaluator (SE) | Residual (SE) |
| --- | --- | --- | --- |
| Patient Care | 0.250 (0.032) | 1.136 (0.127) | 1.851 (0.018) |
| Patient Care: Technical Skills | 0.224 (0.031) | 1.191 (0.137) | 1.792 (0.023) |
| Medical Knowledge | 0.225 (0.035) | 4.738 (0.538) | 5.556 (0.064) |
| Practice Based Learning | 0.220 (0.031) | 1.652 (0.193) | 2.618 (0.032) |
| Professionalism | 0.290 (0.037) | 1.116 (0.124) | 1.857 (0.022) |
| Interpersonal/Communication | 0.252 (0.044) | 2.130 (0.316) | 3.041 (0.062) |
| System Based Practice | 0.211 (0.028) | 1.156 (0.136) | 2.209 (0.023) |
| Overall Assessment | 0.353 (0.043) | 0.905 (0.104) | 1.575 (0.016) |

*Each score was analyzed using a linear mixed-effects model with crossed resident-specific and evaluator-specific random intercepts. The values presented represent the variance estimates and their corresponding standard errors (SE).
