# Supplementary material for: Impact of faculty and resident gender on milestone evaluations in anesthesiology residency: a retrospective analysis
Source: Med Educ Online. 2026 Jul 10;31(1):2688660. doi: 10.1080/10872981.2026.2688660 (PMC13360505; doi:10.1080/10872981.2026.2688660)
Supplement: Supplementary Material — supplemental material.docx [file ZMEO_A_2688660_SM0910.docx]

**Supplemental material**. Example of faculty evaluation of resident for the OB rotation. Each radio button correspond with a numeric score 1-9.
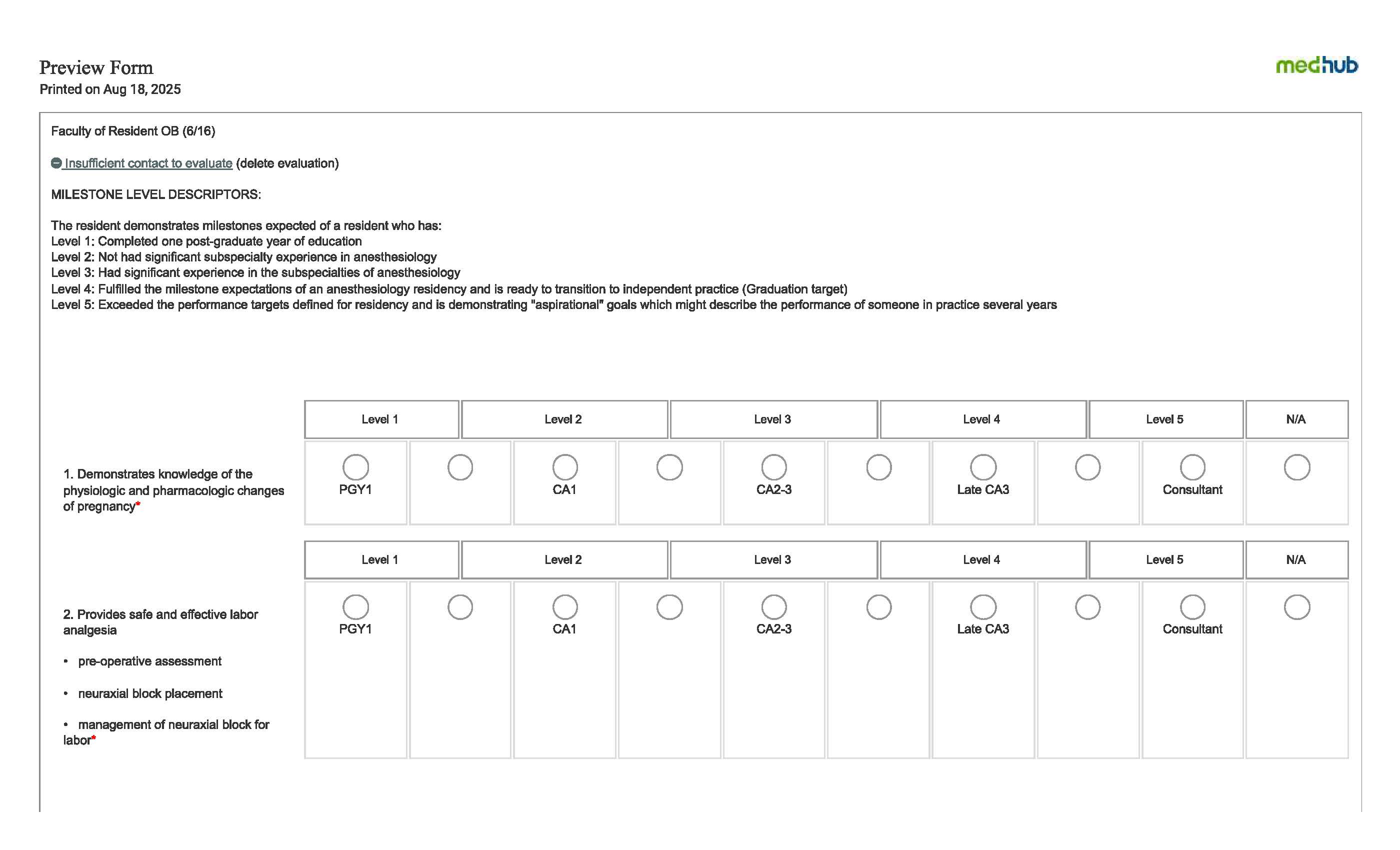

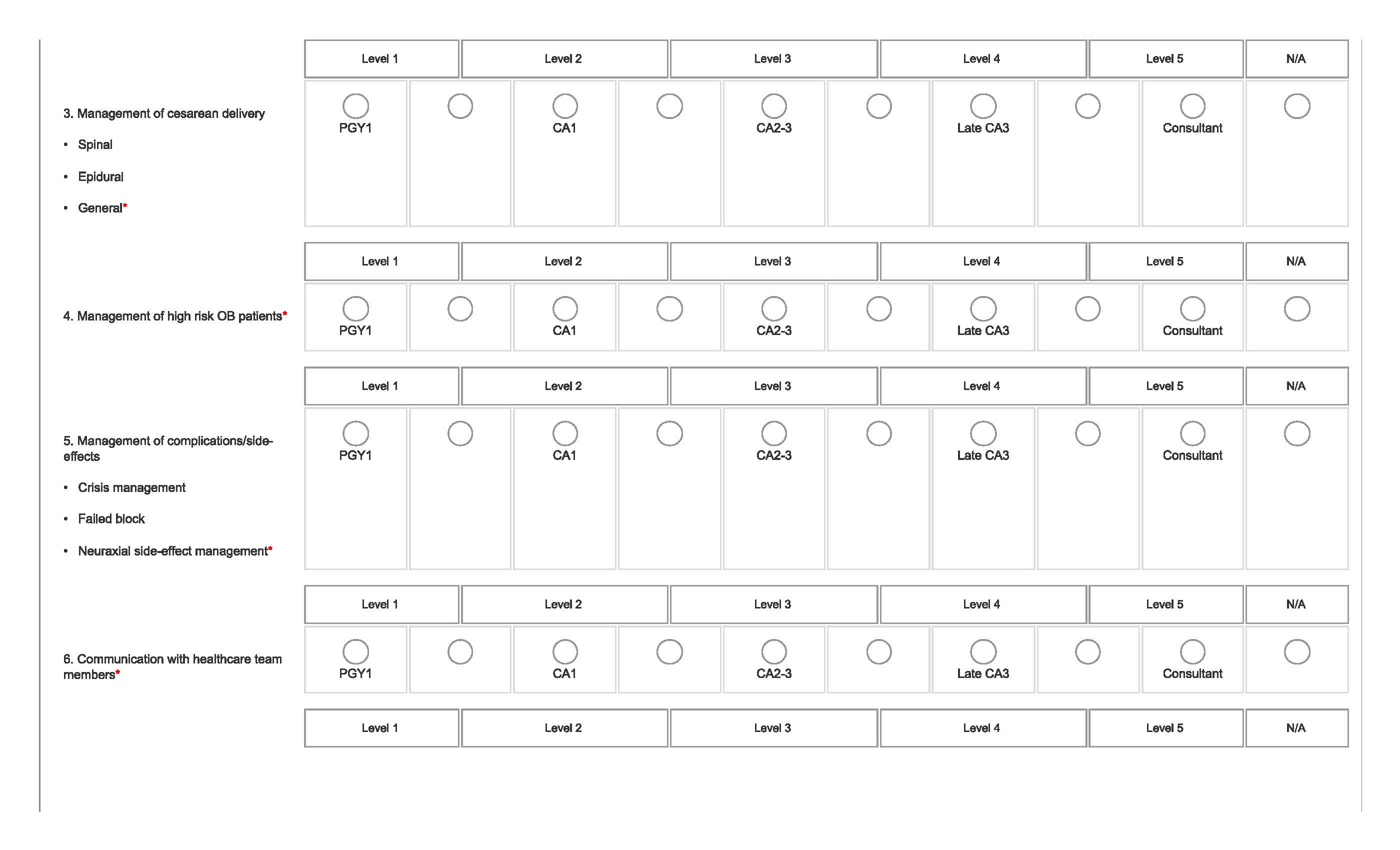

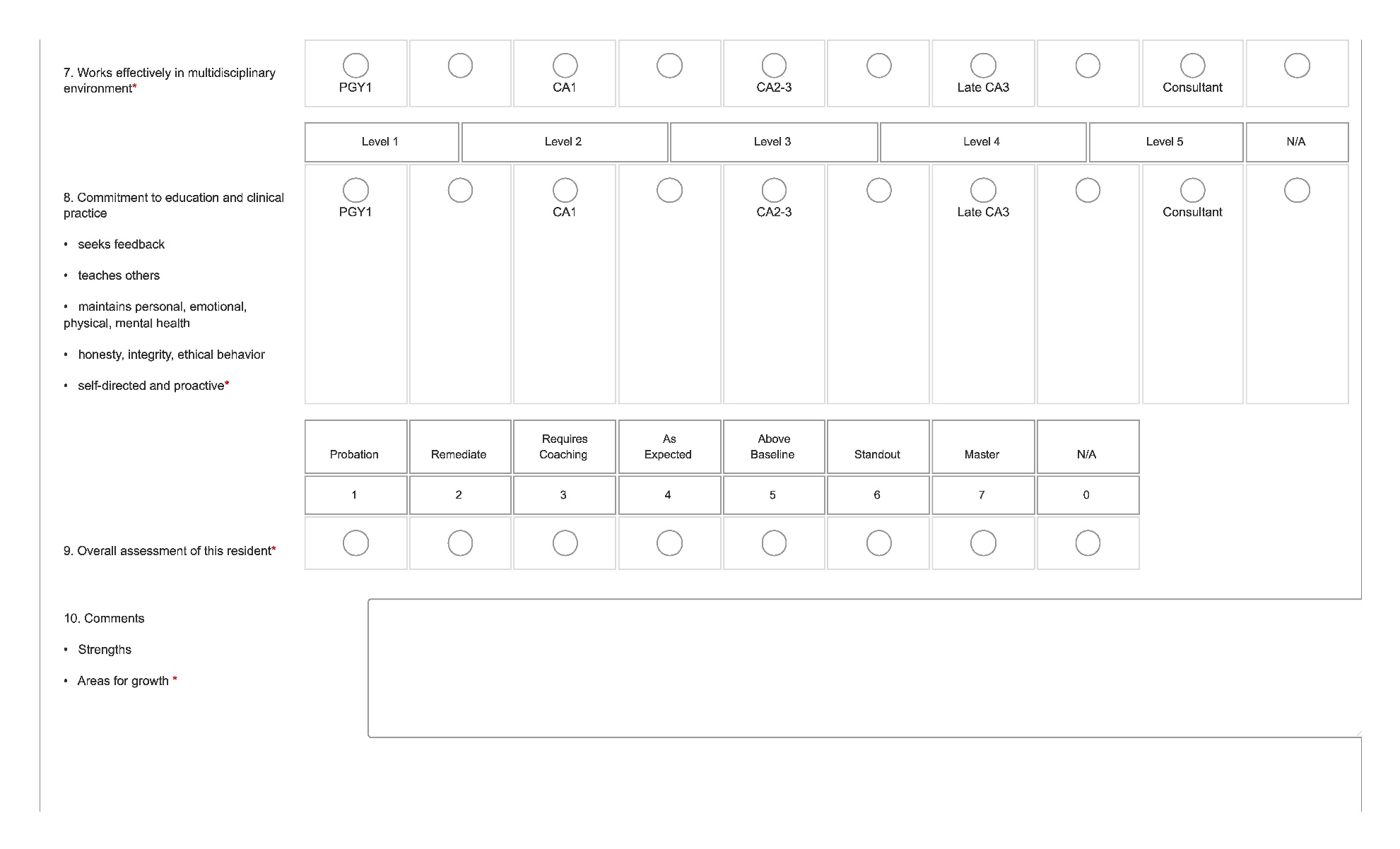

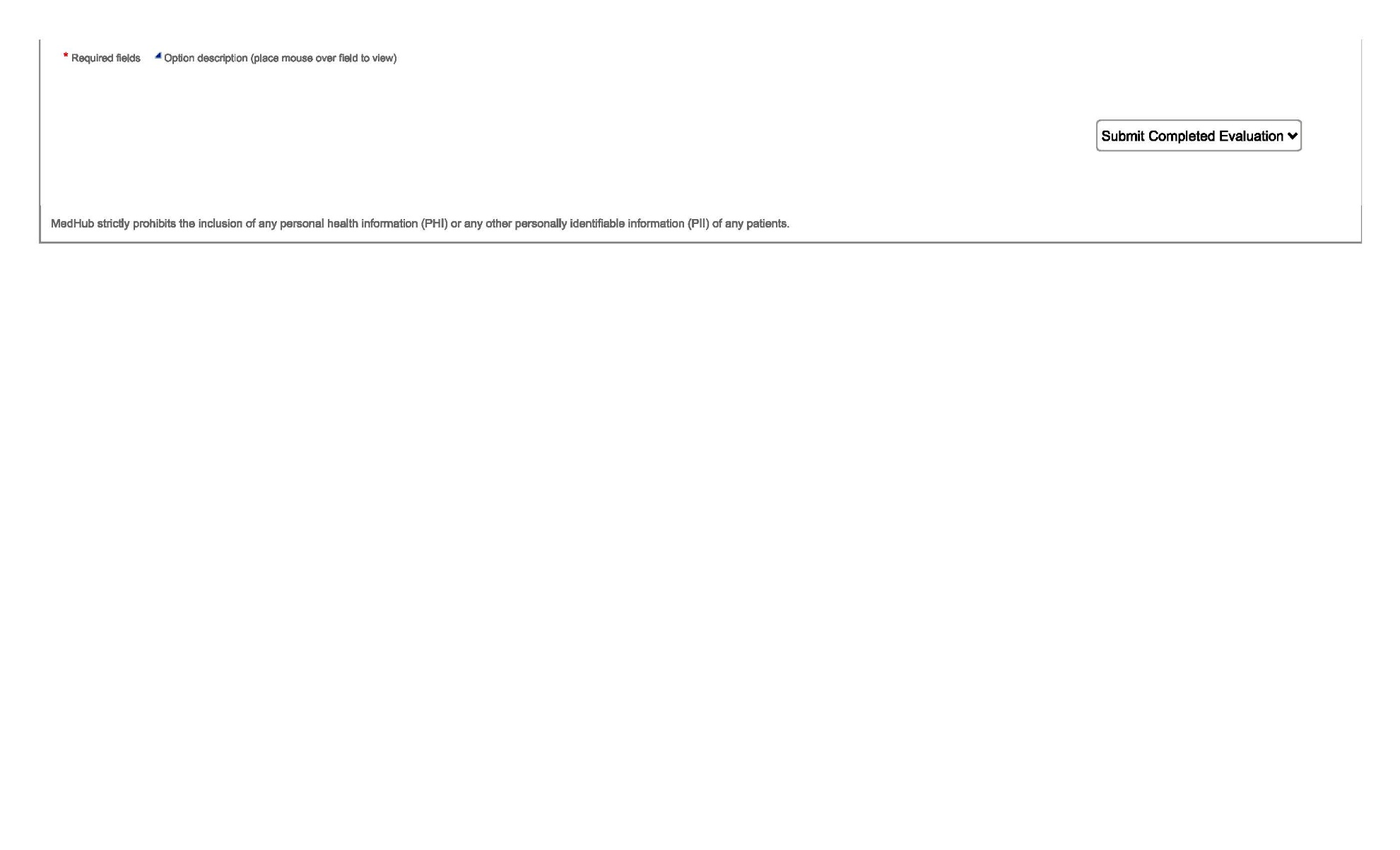


**1 2 3 4 5 6 7 8 9**
